# Cemented in place: kyphoplasty-associated pulmonary cement embolism: a case report

**DOI:** 10.1186/s13256-024-04656-3

**Published:** 2024-08-28

**Authors:** Sung Jun Cho, Hussein Magale, Kiril Dimitrov

**Affiliations:** 1 University of Minnesota Medical School, Minneapolis, MN USA; 2 Internal Medicine Residency, University of Minnesota Medical School, Minneapolis, MN USA; 3 Department of Hospital Medicine, University of Minnesota, Minneapolis, MN USA

**Keywords:** Pulmonary cement embolism, Kyphoplasty, Case report

## Abstract

**Background:**

Kyphoplasty-associated cement extravasation into surrounding tissue and vasculature can lead to life-threatening complications. We present a rare case of significant inferior vena cava cement burden that resulted in pulmonary embolism.

**Case presentation:**

A 74-year-old Caucasian woman with a history of severe osteoporosis, recurrent falls, and spinal compression fracture status post-kyphoplasty of the L4–L5 vertebrae, presents to the emergency department 2 days post-vertebral kyphoplasty due to chest pain, back pain, and dyspnea. Computed tomography of the chest and abdomen showed a metallic density within the inferior vena cava extending superiorly approximately 10 cm from the vertebral L5 level. She was also found to have right lower lobe pneumonia. The patient finished a 10-day course of antibiotics and was discharged home with a 1-month long course of anticoagulation with apixaban per recommendations of a multidisciplinary team consisting of Hematology/Oncology, Interventional Radiology, Vascular Surgery, and Orthopedic Surgery. Unfortunately, the patient was readmitted a month later with shortness of breath. Work up was notable for an influenza type A infection and computed tomography findings of pulmonary cement embolism. The respiratory distress was resolved with supportive care. Despite pulmonary cement burden, the multidisciplinary care team recommended no further anticoagulation. Patient was discharged home with close clinical follow-up and 6 months has since passed at the time of this report without reported complications.

**Conclusions:**

A large cement burden in the inferior vena cava leading to pulmonary cement embolism is a rare event. A high burden of cement predisposes development of pulmonary embolism. A short course of anticoagulation may only be needed for asymptomatic patients.

## Background

Vertebral compression fractures are associated with significant morbidity and mortality. When conservative medical therapy fails, kyphoplasty and vertebroplasty are widely performed procedures for long lasting pain relief [1, 2]. Despite symptom improvement, these procedures are associated with cement extravasation that could lead to life threatening complications. Literature reports cement extravasation has been reported in 30% of kyphoplasty cases (3). Injected cement can leak to nearby anatomical structures including prevertebral soft tissues (in 6–52.5% of the cases), epidural veins (16.5%), spinal canal (37.5%), prevertebral veins (5–16.6%), and the inferior vena cava (4).

Lower risk of cement extravasation is associated with kyphoplasty compared with vertebroplasty (3). The incidence of pulmonary cement embolism (PCE) with kyphoplasty was reported to be 2–26% (5) of the time. There are no validated guidelines for managing pulmonary embolism associated with cement extravasation and leakage into the surrounding vasculature. A systematic review in 2009 recommends no treatment besides clinical follow-up for asymptomatic patients with peripheral PCE and defers to the general guidelines of pulmonary embolism for symptomatic and central PE (6). We present a case of kyphoplasty complication in which a high burden of extravasated cement material leaked into the IVC and eventually resulted in PCE. This case is significant as it demonstrates a rare large burden of IVC cement, clinical evolution of PCE, and sheds light on anticoagulation management.

## Case presentation

A 74-year-old Caucasian woman presented to the hospital emergency department with chest pain, back pain, and dyspnea. Her medical history is significant for chronic obstructive pulmonary disease (COPD), end-stage renal disease (ESRD) on hemodialysis, alcohol-induced liver cirrhosis status post-transplant in 2010, severe osteoporosis, and spinal compression fracture status post-kyphoplasty of the L4–L5 vertebrae 2 days prior to presentation. No pertinent obstetric history. Family history of hypertension and rheumatoid arthritis was noted. She was retired at the time of presentation with no pertinent occupational history. She had a 44 pack-year smoking history and no alcohol use at presentation. Her medications included epoetin alfa 600 unit injection on dialysis days, iron sucrose injection weekly, aspirin 81 mg by mouth daily, lactulose 10 mg/15 mL solution by mouth twice daily, levothyroxine 50 µg tablet by mouth daily, midodrine 5 mg tablets by mouth three times daily, oxycodone 5–10 mg tablet every 4 h as needed for pain, and sertraline 100 mg tablet by mouth daily.

On presentation, the patient's blood pressure was 136/58 mmHg, she had a temperature of 36.8 °C, and she was breathing at 96% oxygen saturation on ambient air. Physical exam was remarkable for reproducible chest pains with sternal pressure and sternal point tenderness. Respiratory exam noted coarse crackles over left lung base. No obvious neurologic abnormalities. No other findings on head/neck, abdominal, skin, or extremity exam. Lab findings on presentation showed elevated serum creatinine of 5.27 mg/dL and BUN of 38.3 mg/dL. Additional findings on metabolic panel showed hypocalcemia of 7.7 mg/dL and hyperphosphatemia of 6.2 mg/dL. Liver function tests showed elevated alkaline phosphatase at 360 U/L, while ALT, AST, and total bilirubin were within normal limits, 12 U/L, 21 U/L, and 0.6 mg/dL respectively. Complete blood count was notable for mild anemia with hemoglobin of 11.2 g/dL, white count of 10.2 10e3/uL, and platelets of 164 10e3/uL. TSH was within normal limits at 3.12uIU/mL. Vitamin D levels were found to be low at 19ug/L (20-75 ug/L).

**Fig. 1:**
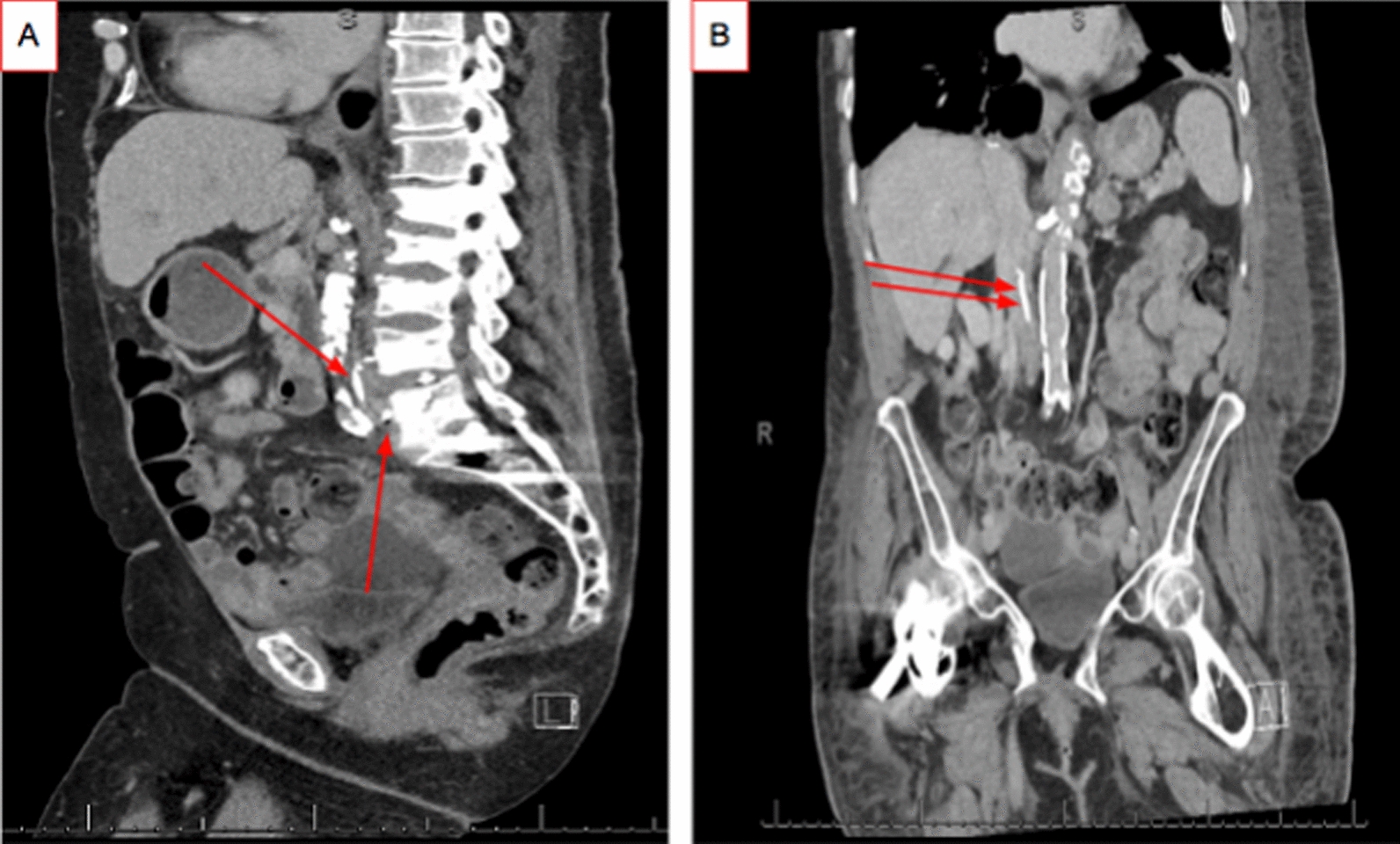
CT abdomen/pelvis, including sagittal (**A**) and coronal (**B**) views with red arrows showing cement extravasation in relation to calcified aorta

Per operative report of kyphoplasty done two days prior to presentation, a trocar was passed into the vertebral bodies of L4 and L5. A balloon inflator was passed via cannula into the vertebral body under fluoroscopy. After balloon inflation, cement was introduced into the vertebral body at no more than 0.5 cc at a time. Fluoroscopy was used to monitor extravasation of cement from the vertebra and none was visualized. Computed tomography (CT) of the abdomen done at presentation showed a metallic density within the inferior vena cava (IVC) extending approximately 10 cm cephalad from the L5 vertebral level (Fig. 1A, B). In addition, she was found to have a right lower lobe pneumonia and was treated initially with intravenous ceftriaxone 1 g every 24 h and per os (PO) azithromycin 500 mg daily. Patient was transitioned to intravenous vancomycin 2.25 and Zosyn 3.375 g/0.375 g every 6 hours for 1 day. She was found to have *Klebsiella pneumonia* sensitive to cefepime and was transitioned to intravenous cefepime 1 g every 12 h for 7 days. A multidisciplinary team, including Interventional Radiology, Hematology/Oncology, and Orthopedic Surgery, was convened given the rarity and potential danger of the complication. The decision was made to start the patient on apixaban 2.5 mg tablets, twice daily for 1 month. Despite the patient's presentation with dyspnea, she did not need any oxygen supplementation and resolved with antibiotics and supportive care.

**Fig. 2:**
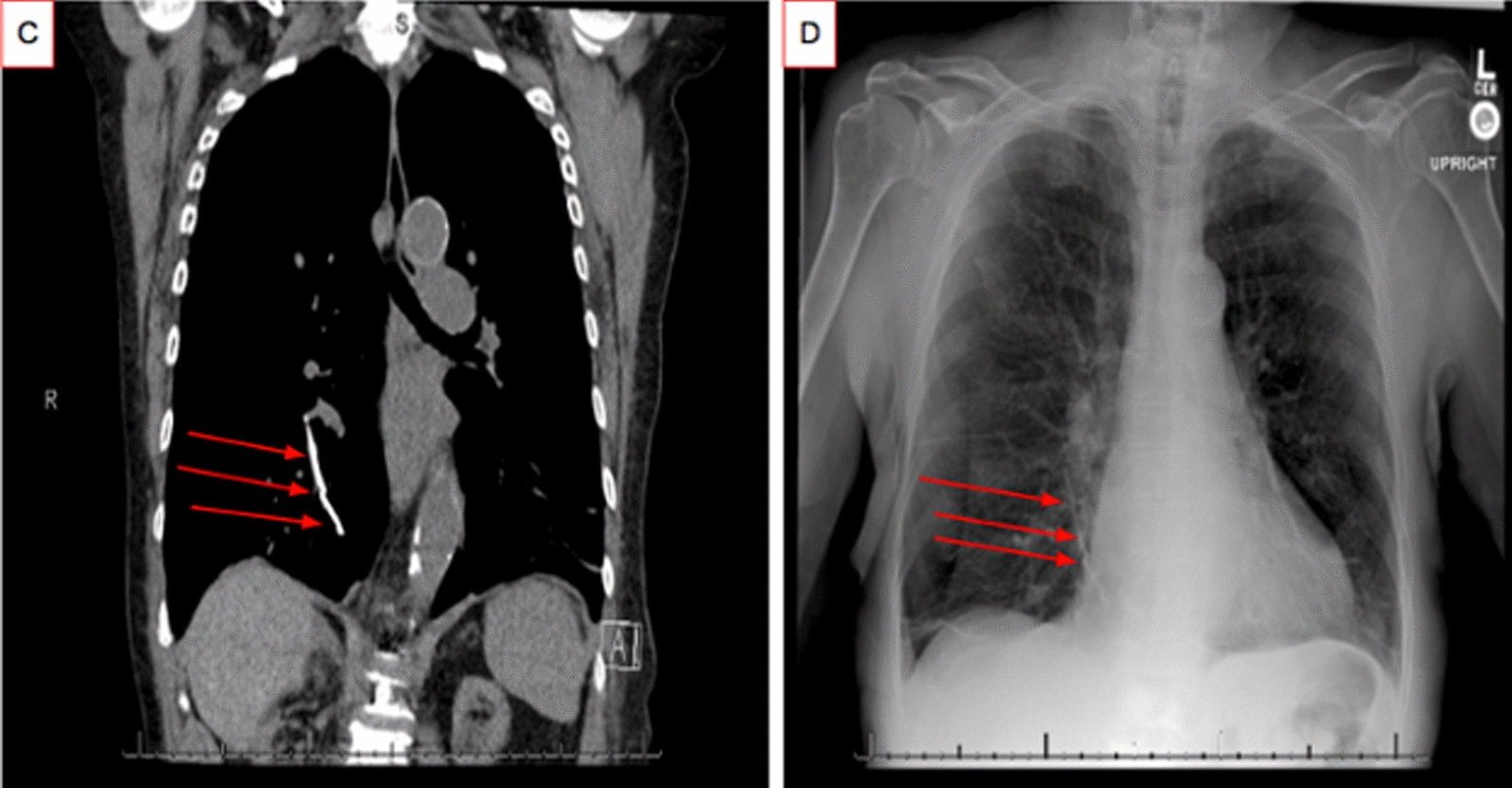
Readmission images demonstrating intravasation of the cement. CT imaging (**C**) and chest x-ray (**D**) showing cement in the right lower lobe

The patient was readmitted approximately 1 month later after being seen in the emergency department with shortness of breath and wheezing. Vitals showed blood pressure was 169/77 mmHg, temperature of 36.5 °C, and 98% oxygen saturation on ambient air. Patient had continued to take all medications from the previous admission. She had finished her course of apixaban prior to admission. Lab results at readmission showed largely similar metabolic panel and complete blood count with hemoglobin of 9.7 g/dL, white blood cell count of 4.4 × 10^3^/dL and platelets of 122 × 10^3^/dL. Work up was notable for a positive influenza type A test. CT demonstrated embolization of the previously identified cement extrusion into the IVC that had lodged in the right lower lobe segmental artery of pulmonary vasculature (Fig. 2C, D). The case was discussed with a multidisciplinary team given the unusual presentation including Orthopedics, Hematology/Oncology, Interventional Radiology, and Vascular Surgery. Hematology recommended against anticoagulation because despite the embolization there is no evidence to suggest an actual thrombin clot and the patient would be a risky candidate for anticoagulation given history of prior falls. Vascular surgery did not feel that there was an indication for mechanical removal of the cement embolus, but did suggest exploring the possibility of an IVC filter given the infrarenal location of remnant cement in the IVC. Interventional Radiology recommended against an IVC filter as this would not prevent further embolization of the cement remaining in her IVC and an IVC filter itself presents a risk for further clots. In discussion with the patient and multidisciplinary team, the final decision was made to follow the patient clinically without anticoagulation. At the time of writing this case presentation, 6 months have since passed without new complications.

## Discussion

To summarize our case, a 74 year-old woman who presented to the ED with chest pain, back pain, and dyspnea was found to have a right lower lobe pneumonia and a cement extravasation into the IVC after kyphoplasty L4–L5 2 days prior to initial presentation. She was treated with intravenous antibiotics and started on oral anticoagulation (apixaban) before discharge. She re-presented to the hospital again 1 month later after finishing the course of anticoagulation for shortness of breath and wheezing and was found to be positive for influenza A and had a CT scan that demonstrated embolization of the previously extravasated cement from her L4–L5 kyphoplasty. After multiple discussions between Vascular Surgery, Interventional Radiology, and Hematology, the decision was made to monitor the patient closely without further anticoagulation. Reports of cement extrusion into the IVC and further into the right atrium, such as this case, are rare and are estimated to occur in 0.5% of cases (6). Literature has also shown that cement embolisms can rarely result in symptomatic PCE and rare incidences of pulmonary embolism from poly(methyl methacrylate) (PMMA) leading to death (7).

Given the increasingly widespread adoption of percutaneous vertebroplasty and kyphoplasty for treatment of osteoporotic vertebral compressions, studying the possible complications of the procedures is imperative. This is an extreme case of extravasation of cement material into the IVC that resulted in a potentially life-threatening pulmonary cement embolism (PCE). Studies have shown that cement material often leaks into the surrounding tissue in 46% of kyphoplasty cases (8).

Treatment for symptomatic individuals often requires the use of anticoagulants for a prolonged period of time. The most common treatment for patients undergoing medical management was heparin followed by 6 months of warfarin (6). In the presented case, given that the patient was asymptomatic, Interventional Radiology recommended one month of oral anticoagulation on apixaban. Given that those suffering from osteoporosis related vertebral fractures, such as this patient, are of advanced age and more likely to have a history of falls, being placed on anticoagulants to treat a PMMA vascular extrusion increases the chances of complications such as bleeding. Surgical management may be indicated if medical approaches are ineffective, but there is little research examining this approach. It is unclear in this case if a surgical approach would have prevented the patient’s PCE.

Another important question this case raises is whether there should be a stronger recommendation of post-procedure imaging. Retrospective studies examining complications from kyphoplasty found PMMA cement embolism in 4–6% of cases, most of which were found incidentally. However, some prospective studies found rates as high as 26% (6, 9). This indicates that PMMA cement pulmonary embolism is most likely under-diagnosed given that most cases are asymptomatic. Having post-procedure imaging done at follow-up can increase the likelihood that PCEs are diagnosed and appropriately managed. Other published cases of PCEs have come to similar conclusions and recommended post-procedure chest x-rays for imaging following vertebroplasty (10). Which imaging modality is considered best for visualization and screening for PCE following procedure still remains unclear. In this case, both CT scans and chest x-rays were done as shown in the images panel. Comparing the two modalities here, the chest x-ray is able to show the cement extravasation similarly to the chest CT images (Fig. 2 C, D). This is consistent with the conclusions of other cases in published literature (11, 12).

Currently there exists a need for greater research into benefits versus harms of post-procedural imaging, either with chest x-ray or CT, after a kyphoplasty. The potential harms of under-diagnosing PMMA cement extravasation included increased the risk of untreated PCE that could result in patient morbidity or mortality. Additionally, with imaging, it is still unclear whether x-ray or CT is a better modality for detecting cement extravasation. There also exists a need to compare vertebroplasty to kyphoplasty as there is little literature to support one procedure over the other. However, there is a noted cost difference between the two. Kyphoplasty can cost 10–20 times more compared with vertebroplasty due to requiring the patient undergo general anesthesia and be admitted overnight for the procedure, along with higher instrumentation costs (13). Additionally, recent meta-analysis found that kyphoplasty may reduce the incidence of cement leakage and have improved vertebral height restoration; however, there generally were no significant differences in terms of measured outcomes between the two, including patient quality of life measures (3, 14). Lastly, there exists a continual need in material science to continue to work on cement material that will reduce the chances of extravasation and PCE.

## Conclusion

This is a rare case of pulmonary cement embolism in a 74-year-old woman. This report illustrates the potential disease course and management of PCEs, along with associated imaging findings. This case raises the question of whether postoperative imaging should be standard of care following kyphoplasty, especially in elderly patients with a history of falls. Anticoagulation with apixaban was given to this patient for one month before discontinuation with no reported adverse events or worsening of symptoms; however, it is unclear if continuing anticoagulation or a more invasive procedure to remove the cement would have prevented the patient’s reported symptoms or worsening of her PCE. Which imaging modality would be best for postoperative monitoring for PCE is a topic of ongoing research. In this presented case, the chest x -ray appeared to be comparable to chest CTs in terms of identifying a PCE (Fig. 2 C, D). Lastly, there exists a continual need to examine and improve our understanding of percutaneous methods of treating vertebral fractures, both in whether one method (kyphoplasty versus vertebroplasty) is superior and whether better materials exist to meet the increasing burden of osteoporosis induced compression vertebral fractures in our aging population.

## Data Availability

The raw data supporting this case will be made available by the authors without undue reservations.
